# Supplementary material for: The Effects of Modified Atmosphere Packaging on the Quality Properties of Water Buffalo Milk’s Concentrated Cream
Source: Molecules. 2023 Jan 30;28(3):1310. doi: 10.3390/molecules28031310 (PMC9920498; doi:10.3390/molecules28031310)
Supplement: Supplementary file 1 [file molecules-28-01310-s001.zip › molecules-2145070-supplementary.pdf]

Table S1. The color characteristics (L*, a*, b*, ΔE) of the CC samples.

	Storage Time (day)				
	Initial sample*	7	14	21	28
			L* value		
MAP1	94,340±0,276^{Aa}	92,581±2,763^{Aa}	91,776±1,222^{Aa}	93,184±0,388^{Aa}	91,508±2,656^{Aab}
MAP2	94,340±0,276^{Aa}	92,694±1,953^{Aa}	93,023±0,648^{Aa}	94,251±0,692^{Aa}	93,793±0,854^{Aa}
Control	94,340±0,276^{Aa}	93,468±1,784^{ABa}	90,815±0,502^{ABCa}	89,322±1,431^{BCb}	87,380±0,255^{Cb}
			a* value		
MAP1	-2,978±0,025^{Aa}	-3,262±0,295^{Aa}	-3,209±0,254^{Aa}	-3,164±0,265^{Aa}	-3,110±0,229^{Ab}
MAP2	-2,978±0,025^{Aa}	-3,244±0,136^{Aa}	-3,258±0,111^{Aa}	-3,180±0,137^{Aa}	-3,062±0,061^{Ab}
Control	-2,978±0,025^{Aa}	-3,462±0,021^{Aa}	-2,218±1,299^{Aa}	-1,587±0,825^{Aa}	0,168±1,586^{Aa}
			b* value		
MAP1	5,58±0,33^{Ba}	6,22±0,57^{Bb}	8,57±0,39^{Ab}	8,66±0,43^{Ab}	9,15±0,14^{Ab}
MAP2	5,58±0,33^{Ca}	7,48±0,06^{Ba}	8,48±0,30^{ABb}	8,22±0,34^{ABb}	9,05±0,42^{Ab}
Control	5,58±0,33^{Ca}	7,68±0,16^{Ca}	15,96±0,88^{Ba}	21,98±1,00^{Aa}	25,16±0,14^{Aa}
			ΔE value		
MAP1	0	2,322±1,283^{Ba}	3,999±0,559^{Bb}	3,184±0,230^{Db}	4,717±1,807^{Ab}
MAP2	0	2,911±0,074^{Ca}	3,092±0,565^{Ba}	2,487±0,251^{DEb}	3,389±0,211^{ABb}
Control	0	2,725±0,896^{Ca}	11,099±0,767^{Ca}	17,075±1,474^{Ba}	20,704±1,880^{Aa}

* The initial sample was analyzed before any application.

$^{A-B}$ For each application, different superscript uppercase letters show differences between the samples (P < 0.05).

$^{a-b}$ Different superscript lowercase letters show differences between the storage times (P < 0.05).

Table S2. Fatty acid compositions (%) of CC samples (mean ± standard deviations, n = 2).

	**Initial sample***		**28th day****
**C4**	4,129±0,161	MAP1	3,924±0,010^{A}
		MAP2	3,916±0,026^{A}
		Control	3,829±0,014^{B}
**C6**	2,083±0,079	MAP1	1,991±0,005^{A}
		MAP2	1,998±0,010^{A}
		Control	1,968±0,017^{A}
**C8**	1,094±0,041	MAP1	1,052±0,004^{A}
		MAP2	1,059±0,002^{A}
		Control	1,035±0,005^{B}
**C10**	2,021±0,063	MAP1	1,962±0,001^{AB}
		MAP2	1,978±0,009^{A}
		Control	1,944±0,007^{B}
**C12**	2,617±0,069	MAP1	2,573±0,006^{A}
		MAP2	2,569±0,017^{A}
		Control	2,573±0,021^{A}
**C14**	12,304±0,234	MAP1	12,354±0,098^{A}
		MAP2	12,447±0,022^{A}
		Control	12,500±0,006^{A}
**C14.1**	0,890±0,014	MAP1	0,892±0,006^{A}
		MAP2	0,892±0,004^{A}
		Control	0,860±0,004^{B}
**C15**	1,544±0,006^{b}	MAP1	1,568±0,001^{Aa}
		MAP2	1,573±0,008^{Aa}
		Control	1,573±0,003^{Aa}
**C16**	35,233±0,187^{b}	MAP1	35,594±0,233^{A}
		MAP2	35,357±0,497^{A}
		Control	36,115±0,122^{Aa}
**C16.1**	2,962±0,020	MAP1	2,930±0,001^{B}
		MAP2	2,976±0,013^{A}
		Control	2,918±0,008^{B}
**C17**	0,852±0,002	MAP1	0±0
		MAP2	0±0
		Control	0±0
**C17.1**	0,375±0,001	MAP1	0,372±0,001^{B}
		MAP2	0,379±0,001^{A}
		Control	0,371±0,001^{B}
**C18**	8,272±0,077^{b}	MAP1	8,528±0,074^{A}
		MAP2	8,590±0,048^{Aa}
		Control	8,698±0,034^{Aa}
**C18.1 T**	1,205±0,010	MAP1	1,256±0,021^{A}
		MAP2	1,343±0,154^{A}
		Control	1,338±0,145^{A}
**C18.1 C**	17,949±0,112	MAP1	17,938±0,014^{B}
		MAP2	18,213±0,004^{A}
		Control	17,778±0,062^{C}
**C18.2 T**	0,144±0,000^{a}	MAP1	0,144±0,001^{A}
		MAP2	0,141±0,003^{A}

		Control	$0,138±0,001^{Ab}$
**C18.2 C**	$1,068±0,007^{a}$	MAP1	$1,054±0,011^{A}$
		MAP2	$1,077±0,021^{A}$
		Control	$1,034±0,006^{Ab}$
**C18.3**	$0,210±0,004^{b}$	MAP1	$0,221±0,003^{Ba}$
		MAP2	$0,221±0,000^{Ba}$
		Control	$0,232±0,002^{Aa}$
**C18.3 N6**	0±0	MAP1	$0,123±0,001^{A}$
		MAP2	$0,120±0,001^{B}$
		Control	$0,117±0,000^{C}$
**C20.1**	$0,411±0,002^{a}$	MAP1	$0,403±0,001^{Ab}$
		MAP2	$0,408±0,001^{A}$
		Control	$0,390±0,004^{Bb}$
**C20.3 N3**	0,116±0,00	MAP1	$0,127±0,002^{A}$
		MAP2	$0,125±0,002^{A}$
		Control	$0,142±0,014^{A}$
**C20.4**	$0,175±0,037^{a}$	MAP1	$0,111±0,004^{A}$
		MAP2	$0,071±0,008^{Bb}$
		Control	$0,077±0,002^{Bb}$
**C20.5**	0,007±0,009	MAP1	$0,014±0,000^{A}$
		MAP2	$0,019±0,001^{A}$
		Control	$0,017±0,003^{A}$
**C24**	$0,059±0,001^{a}$	MAP1	$0,059±0,001^{A}$
		MAP2	$0,059±0,001^{A}$
		Control	$0,056±0,000^{Bb}$
**SFA**	70,205±0,763	MAP1	$69,603±0,394^{A}$
		MAP2	$69,543±0,354^{A}$
		Control	$70,290±0,187^{A}$
**UFA**	25,511±0,084	MAP1	$25,581±0,037^{A}$
		MAP2	$25,981±0,169^{A}$
		Control	$25,410±0,237^{A}$
**MUFA**	23,792±0,115	MAP1	$23,789±0,027^{A}$
		MAP2	$24,209±0,139^{A}$
		Control	$23,654±0,225^{A}$
**PUFA**	1,719±0,031	MAP1	$1,792±0,010^{A}$
		MAP2	$1,772±0,030^{A}$
		Control	$1,756±0,012^{A}$

* The initial sample was analyzed before any application. ** FA analysis values of MAP1, MAP2, and Control samples at the end of storage.

$^{A-B}$ For each application, different superscript uppercase letters show differences between the samples ($P < 0.05$).

$^{a-b}$ Different superscript lowercase letters show differences between the storage times ($P < 0.05$).

If there was no statistical difference, lowercase and uppercase superscript were not used.

SFA: Saturated fatty acids, MUFA: Mono-unsaturated fatty acids, PUFA: Poly-unsaturated fatty acids.

Table S3. Free fatty acid concentrations (mg/kg) of CC samples (mean ± standard deviations, n = 2).

	Initial sample*		28th day**
**C:4**	0^{b}	MAP1	$3,00±1,57^{Ba}$
		MAP2	0^{B}
		Control	$70,31±14,40^{Aa}$
**C:6**	0^{b}	MAP1	$3,40±0,27^{Ba}$
		MAP2	0^{B}
		Control	$19,16±2,80^{Aa}$
**C:8**	0^{b}	MAP1	$6,85±0,60^{B}$
		MAP2	0^{B}
		Control	$27,30±6,80^{Aa}$
**C:10**	$0,94±0,04^{b}$	MAP1	$32,75±4,60^{Ba}$
		MAP2	$20,29±4,06^{Ba}$
		Control	$294,87±5,44^{Aa}$
**C:12**	$26,73±7,50^{b}$	MAP1	$39,29±5,71^{B}$
		MAP2	$25,91±2,44^{B}$
		Control	$359,62±2,72^{Aa}$
**C:14**	$88,61±21,38^{b}$	MAP1	$110,67±9,42^{B}$
		MAP2	$63,09±3,30^{C}$
		Control	$1041,03±9,07^{Aa}$
**C:15**	0^{b}	MAP1	0^{B}
		MAP2	0^{B}
		Control	$97,44±10,88^{Aa}$
**C:16**	$459,72±39,34^{b}$	MAP1	$513,40±15,00^{B}$
		MAP2	$347,93±9,08^{B}$
		Control	$2203,85±81,59^{Aa}$
**C:16.1**	0^{b}	MAP1	$57,13±9,71^{Ba}$
		MAP2	$17,52±9,58^{C}$
		Control	$722,44±2,72^{Aa}$
**C:18**	$193,50±10,34^{b}$	MAP1	$233,62±3,55^{Ba}$
		MAP2	$139,28±0,10^{Cc}$
		Control	$639,10±24,48^{Aa}$
**C:18.1**	$172,49±43,03^{b}$	MAP1	$490,79±82,48^{Ba}$
		MAP2	$146,07±16,19^{B}$
		Control	$5892,31±137,80^{Aa}$
**C:18.2**	0^{b}	MAP1	$24,69±5,31^{Ba}$
		MAP2	0^{C}
		Control	$241,03±3,63^{Aa}$
**SFFA**	$791,09±79,88^{b}$	MAP1	$942,75±33,38^{B}$
		MAP2	$596,50±13,90^{C}$
		Control	$4752,67±64,84^{Aa}$
**MUFFA**	$172,49±43,03^{b}$	MAP1	$547,92±92,20^{Ba}$
		MAP2	$163,59±25,76^{B}$
		Control	$6614,74±140,52^{Aa}$
**PUFFA**	0^{b}	MAP1	$24,69±5,31^{Ba}$
		MAP2	0^{C}
		Control	$241,03±3,63^{Aa}$

* The initial sample was analyzed before any application. ** FFA analysis values of MAP1, MAP2, and Control samples at the end of storage.
$^{A-B}$ For each application, different superscript uppercase letters show differences between the samples (P < 0.05).

$^{a-b}$ Different superscript lowercase letters show differences between the storage times ($P < 0.05$).
If there was no statistical difference, lowercase and uppercase superscript were not used.
SFFA: Saturated free fatty acids, MUFFA: Mono-unsaturated free fatty acids, PUFFA: Poly-unsaturated free fatty acids.
